# Nigerian stakeholders’ perceptions of a pilot tier accreditation system for Patent and Proprietary Medicine Vendors to expand access to family planning services

**DOI:** 10.1186/s12913-022-08503-3

**Published:** 2022-09-03

**Authors:** Funmilola M. OlaOlorun, Aparna Jain, Emily Olalere, Ene Daniel-Ebune, Kayode Afolabi, Emeka Okafor, Sara Chace Dwyer, Osimhen Ubuane, Toyin O. Akomolafe, Sikiru Baruwa

**Affiliations:** 1 University of Ibadan, Ibadan, Nigeria; 2 Population Council, Washington D.C., USA; 3 Pharmacists Council of Nigeria, Abuja, Nigeria; 4 Federal Ministry of Health, Abuja, Nigeria; 5 Society for Family Health, Abuja, Nigeria; 6 Population Council, Abuja, Nigeria

**Keywords:** Family planning services, Tier accreditation system, Task shifting, Private sector

## Abstract

**Background:**

Community Pharmacists (CPs) and Patent and Proprietary Medicine Vendors (PPMVs) are often the first point of care for many Nigerians, and when sufficiently trained, they contribute to the expansion of family planning services. Nigeria’s task shifting policy and existing regulatory and licensing bodies provide the enabling environment for PPMVs to be stratified and trained. This study explored the perceptions of stakeholders toward the pilot three-tier accreditation system instituted by the Pharmacists Council of Nigeria with support from the IntegratE project.

**Methods:**

Two rounds of qualitative phone interviews were conducted among stakeholders in Kaduna and Lagos states in 2020 and 2021. In addition, there were two rounds of phone interviews with CPs and PPMVs (program recipients) from Lagos and Kaduna states. All participants were purposively selected, based on their involvement in the IntegratE project activities. Interviews were recorded, transcribed, and coded using Atlas.ti software. Thematic analysis was conducted.

**Results:**

Fifteen stakeholders and 28 program recipients and 12 stakeholders and 30 program recipients were interviewed during the first and second rounds of data collection respectively. The data are presented around three main themes: 1) the pilot three-tier accreditation system; 2) enabling environment; and 3) implementation challenges. The accreditation system that allows for the stratification and training of PPMVs to provide family planning services was perceived in a positive light by majority of participants. The integrated supportive supervision team that included representation from the licensing and regulatory body was seen as a strength. However, it was noted that the licensing process needs to be more effective. Implementation challenges that need to be addressed prior to scale up include bottlenecks in licensing procedures and the deep-rooted mistrust between CPs and PPMVs.

**Conclusion:**

Scale up of the three-tier accreditation system has the potential to expand access to family planning services in Nigeria. In other resource-poor settings where human resources for health are in short supply and where drug shops are ubiquitous, identifying drug shop owners, training them to offer a range of family planning services, and providing the enabling environment for them to function may help to improve access to family planning services.

**Supplementary Information:**

The online version contains supplementary material available at 10.1186/s12913-022-08503-3.

## Background

Over the years, while many countries have made significant progress in achieving the 5th Sustainable Development Goal to “achieve gender equality and empower all women and girls”, Nigeria has made only little progress [1]. In order to ensure universal access to sexual and reproductive health and rights by 2030, as stated in the sixth target of this goal, innovative approaches to scale service delivery must be considered. This poses an added challenge in Nigeria where huge shortages in human resources for health exist.

The task-shifting (TS) approach proposed by the World Health Organization (WHO), is intended to strengthen and expand the health workforce, and increase access to health care services, including family planning (FP) [2]. In Nigeria, this public health initiative is being used as a stopgap to address the lingering problem of widespread shortages in human resources for health. In shifting tasks from more highly qualified to less specialized health care providers, Nigeria could efficiently use the health workforce to improve access to FP services. The implementation of Nigeria’s Task Shifting and Task Sharing policy [3] enables the private sector to support the government by training Community Pharmacists (CPs) and Patent and Proprietary Medicine Vendors (PPMVs) to offer FP services. Involvement of CPs and PPMVs in FP service delivery is evidence-based and has been identified as a high-impact practice [4].

Community pharmacists are formally trained and have completed at least an undergraduate degree in pharmacy from an approved Faculty/School of Pharmacy in Nigeria or oversees. CPs provide pharmaceutical care, pharmaceutical services and primary health care services in pharmacies to the communities that they serve. PPMVs, on the other hand, own drug shop outlets and do not have any formal educational requirements. They are licensed by the Pharmacists Council of Nigeria (PCN) to sell a limited number of pre-packaged, over-the-counter medicines and medical products, but prohibited from selling prescription medications (e.g., antibiotics) or conducting invasive medical procedures. PCN is a Federal Government parastatal established by an Act in 2004 to regulate and control pharmacy education, training and practice [5]. Recent research has noted the need for PCN to improve on their regulatory role, also citing the challenge they face regarding the lack of sufficient human and financial sources for regulation [6].

CPs and PPMVs often serve as the first point of care for many Nigerians, especially those in rural and hard-to-reach areas. Their ubiquitous geographic distribution in Nigeria and other sub Saharan African countries uniquely positions them to support the expansion of access to primary health care and FP services [7–9]. In Nigeria, 1 in 5 modern contraceptive users received their last method from a PPMV, and 1 in 10 from a CP [10]. Notably, younger women (15–24 years) were more likely to report obtaining their last method from a PPMV, while older women (25–49 years) were more likely to have obtained their method from a CP [11]. Apart from their ubiquity and proximity, the advantages offered by pharmacies and drug shops in the private sector include their flexible and extended hours, and their personable interactions with their clients [7]. Furthermore, unlike the public sector, CPs and PPMVs do not typically charge separate consultation fees [8].

### The IntegratE project

The IntegratE project, is a four-year initiative (2017–2021) funded by the Bill & Melinda Gates Foundation & Merck Sharp & Dohme for Mothers, and implemented in Nigeria by Society for Family Health, the consortium lead, working with Marie Stopes International, Planned Parenthood Federation of Nigeria, Population Council, and PharmAccess. The goal of the project was to increase access to contraceptive methods in Lagos and Kaduna States of Nigeria by collaborating with CPs and PPMVs, as well as with their regulatory body, the PCN to jointly establish a sustainable regulatory mechanism that supports CPs and PPMVs to offer high-quality FP services. One of IntegratE’s project activities included a pilot three-tier accreditation system for PPMVs based on their healthcare qualifications. In this system, PPMVs are classified into three tiers based on whether or not they have healthcare qualifications. Table 1 describes the three tiers and the training received by each tier. CPs are outside of the accreditation system. More information about IntegratE and its activities are available at https://integrateeproject.org.ng [12].

**Table 1:** Description of the three-tier accreditation system

Provider type	Description	Training received
Tier 1 PPMVs	PPMVs without healthcare qualifications	• FP counseling and referral • Refill of oral contraceptives
Tier 2 PPMVs	PPMVs with healthcare qualifications	• FP counseling and referral • Provision of oral contraceptives • Injectable administration • Implant insertion and removal
Tier 3 PPMVs	PPMVs who are also pharmacy technicians	• Same as Tier 2
CPs	Pharmacy Professionals with Degree in B.Pharm/ Pharm.D. **Outside of accreditation system**	• FP counseling and referral • Provision of oral contraceptives • Injectable administration • Implant insertion and removal

While considering the adoption of a tier accreditation system, PCN conducted a study tour to learn from another accreditation program in Tanzania that also involved drug shop owners. The Tanzania Food and Drugs Authority worked with Management Sciences for Health to design an accredited drug dispensing outlet (ADDO) program to improve access to high quality, affordable medicine and pharmaceutical services, particularly in rural and peri-urban areas. The ADDO program involved training and supervising drug shop owners, while providing incentives and ensuring appropriate referrals and regulatory enforcement. The program was piloted in 2003 and by 2013, it had been scaled up throughout mainland Tanzania. Services offered by the retail drug shop owners were expanded to include primary health care services, including maternal and child health and family planning services. Management Sciences for Health was involved in transferring and replicating the ADDO model in Uganda and Liberia, while adapting to the specific country contexts [8, 9].

### The three-tier accreditation system

PPMVs are a very heterogeneous group, ranging from those who can just read and write, to those with just secondary school education to nurse/midwives who have retired from government institutions after decades of service. This heterogeneity presents a group of individuals who can be engaged to scale basic healthcare services, especially in rural communities where highly skilled healthcare workers are few and far between. In line with Nigeria’s TS policy, PCN is considering stratifying PPMVs in line with their healthcare qualifications (See Table 1). The three-tier accreditation system serves as a pilot test-run of this plan. The components of the accreditation system include: (1) Mandatory Entry-Point Training Program (MEPTP), consisting of 4 weeks of formal training using standardized training manuals and facilitator guides; (2) accreditation/inspection of the patent medicine shop of the PPMV; (3) standardization of the patent medicine shop with appropriate branding and signage; (4) developing capacity of PPMVs for referrals. Although CPs are outside of the accreditation system, PCN plans to involve them to provide mentorship and supportive supervision to PPMVs through the hub-and-spoke model where a CP (the hub) oversees the activities of several PPMVs (the spokes). However, this phase of the pilot program is yet to take off.

The three-tier accreditation system was designed with the WHO TS guidelines in mind. The WHO guidelines include identifying and engaging stakeholders from the beginning, obtaining national endorsement for adoption of TS, and ensuring an enabling regulatory framework. The regulatory role of PCN in managing the activities of CPs and PPMVs, and ensuring they remain within their scope of practice is in keeping with these WHO guidelines. The inception phase of the IntegratE project included wide consultations with professional and regulatory bodies. PCN also clearly detailed the expected competencies and scope of practice for CPs and PPMVs who are trained to offer FP services. In order to encourage sustained high-quality services, WHO also recommended “supportive supervision and clinical monitoring” on a regular basis within existing structures [2].

The WHO guidelines speak to important elements that assure quality of care, such as, “standardized training” and “certification and assessment”. Under the pilot three-tier accreditation system, PPMVs received standardized FP training to offer FP services, but the specific type of FP method they can offer is based on their tier. Formal educational training support for PPMVs was conducted through the Mandatory Entry Point Training Program (MEPTP) designed in conjunction with key government stakeholders. This training was integrated into the existing and accredited Schools and Colleges of Health Technology (SCHT) to support sustainability and is tied to “certification, registration, and career progression”, as recommended by WHO [2]. SCHT are formal training institutions accredited by the Nigerian Board for Technical Education for building technical and vocational skills to address manpower gaps in Nigeria. These schools may be publicly or privately owned, and run a variety of certificate and diploma programs.

PCN developed a training curriculum, scope of practice, and premises standard for each of the three tiers of PPMVs. The training curriculum is the same across all training institutions. Each tier is permitted a specified scope of practice, including drugs they can stock. There are documented standards for locating a new patent medicine shop, such as ensuring it is at least 400 m away from any pharmaceutical premises, and at least 200 m away from a registered patent medicine shop. The accreditation process is in two phases: Personnel (training) accreditation and Premises accreditation. It is expected that to achieve full accreditation, the vendor must meet the minimum requirements for both the training and premises standards. Personnel accreditation involves the completion of PCN prescribed MEPTP by the vendor. The minimum requirement is a score of 50% in the end-of-program examination. After this has been attained, the premises accreditation procedure can be initiated.

For the premises accreditation, the vendor applies to PCN, requesting for his/her premises to be inspected. PCN engages and trains pharmaceutical inspectors who are conversant with all the operating standards for PPMVs to visit such premises. During the inspection, the inspectors use a standardized inspection reporting form, including an accreditation checklist to ensure uniformity of standards across premises and across states. The checklist assesses the size of the premises, the storage conditions of the drugs, availability of counselling area, presence of a hand wash basin, availability of a refrigerator, proper lighting system, proper arrangement of drugs according to disease group, availability of waste bins and sharps boxes (for Tier 2 and Tier 3 PPMVs providing injectable contraceptives). For a successful premises inspection, the vendor must meet the size and space standards, and have in place all the facilities listed above at the time of the inspection. Once the personnel and premises accreditation standards are met, PCN issues an accreditation certificate and branded identification signage which differs from one tier to the other. This differentiates those that have been accredited from those that have not been accredited.

To ensure sustainability, the WHO guidelines recommend that financial and non-financial incentives should be used to retrain staff, and to enhance their performance to ensure a sustainable program. Furthermore, the guidelines recommend that health workers should receive commensurate wages and that TS should be appropriately costed, and adequately financed by the government. Moreover, for proper organization of services, the WHO guidelines recommend adapting the TS practice to the unique context, and ensuring efficient referral services are in place. In the Nigerian context, PPMVs are in the private sector, and thus different from lower cadre government workers who would receive a salary. PPMVs generate their own income through sales of goods and services in their patent medicine or drug shops. Their financial incentive comes from their business where they should make a profit. This profit makes them more willing to pay for training that is compulsory for them to keep their doors open. In the pilot program, training costs were paid for by the donor-supported IntegratE program. The inbuilt sustainability plan for the tier accreditation involves using existing SCHT for training PPMVs. The institutionalization of training was identified in the Tanzanian ADDO project as a “critical step towards independence from donor funding” [9]. It is expected that this tier accreditation model will be self-financing since PPMVs will have to pay for their own training. A non-financial incentive that the PPMVs will enjoy as part of the tier accreditation system is a branded logo. In the eyes of the public seeking services, this branding will give PPMVs credibility and show that they are recognized by the government.

Though innovative and beneficial, the pilot three-tier accreditation system has challenges that need to be carefully assessed to allow for workable solutions to emerge. In order to recommend the scale-up of this novel approach for expanding access to FP services in Nigeria, it is essential to understand the perceptions of different groups of stakeholders regarding the pilot three-tier accreditation system, and to incorporate these into more objective program measures. This paper is focused on the perceptions of a variety of stakeholders toward PCN’s pilot three-tier accreditation system. The stakeholders include leaders of the Association of Community Pharmacists of Nigeria (ACPN); leaders of National Association of Patent and Proprietary Medicine Dealers (NAPPMED); PCN officials; and Ministry of Health (MoH) officials in Lagos and Kaduna states. In order for the three-tier accreditation system to function efficiently and to be sustainable, the right enabling environment must be put in place. The IntegratE project has built on the private sector advantage that CPs and PPMVs already have, and continues to seek ways to ensure appropriate and adequate regulation of these healthcare providers by working closely with PCN, their regulatory body. The purpose of this study is to explore and describe the perceptions of stakeholders regarding PCN’s pilot three-tier accreditation system implemented with support from the IntegratE project. Understanding stakeholders’ perceptions of the accreditation system is paramount as the sustainability of the program depends heavily on the engagement and support of stakeholders at PCN, the Federal and State Ministries of Health, and the respective professional associations.

## Methods

### Study design and context

This qualitative study was conducted in Nigeria, Africa’s most populous nation. Nigeria runs a three-tier health care system with delivery of health care services through the public sector at the primary, secondary, and tertiary levels. Primary level facilities include health posts, health clinics, and primary health centers, while secondary or referral facilities include general hospitals and comprehensive health centers. Referral of patients and clients is generally from the primary to secondary to tertiary, or specialist levels, the latter consisting of university teaching hospitals and federal medical centers, but should ideally be bi-directional. Patients and clients can also seek services in the private sector that includes private hospitals and clinics, faith-based facilities, community pharmacies, and patent medicines shops.

The study was conducted in two of the 36 states in Nigeria: Kaduna in the north, and Lagos in the south. These two states were purposively selected for the convenience of the donors in order to consolidate other existing investments through other projects and organizations (Nigerian Urban Reproductive Health Initiative, Planned Parenthood Federation of Nigeria, Association for Reproductive and Family Health) in these two states, and to build on existing partnerships. PCN’s three-tier accreditation model was first piloted in Kaduna and Lagos states as a collaborative venture with the IntegratE project that is implementing the model.

### Sampling and recruitment

Fifteen (15) and 12 stakeholders respectively were interviewed by phone between October and December, 2020 and June and July 2021. Stakeholders were purposively selected based on the leadership positions they held in groups, organizations and institutions involved in the implementation of the pilot three-tier accreditation system. It was anticipated that saturation would be attained by interviewing 2–3 stakeholders from each group, organization or institution. Those interviewed included CP and PPMV leaders from their respective associations, officials at the State MOH, Federal MOH, and PCN.

### Data collection

While the pilot three-tier accreditation system was still in the early implementation stages during the first round of interviews, implementation was in full bloom during the second round of interviews. A semi-structured in-depth interview guide was used to obtain information from each group of respondents. The guides used were drafted by an expert at the Population Council, based on the study objectives, and then shared internally for additional feedback. Face validity was further conducted by sharing with a Consultant content expert who met with the team virtually to discuss the tools, leading to further refinement. The tools were piloted on individuals who were familiar with the accreditation system, but not part of the population from which the study sample was drawn. The interview guide covered much more than the three-tier accreditation which is the scope of the current paper.

Topics discussed with stakeholders relevant to this manuscript included: thoughts on Nigeria’s TS policy; experiences with supportive supervision; knowledge on how CPs & PPMVs are licensed and supervised; knowledge and perception of three-tiered accreditation system; perceived effects of accreditation system on family planning services; implementation challenges of accreditation system, and how to overcome them; and expectations of accreditation system. The semi-structured interview guide was similar in both rounds, but with more emphasis on actual implementation activities and challenges during the second round of stakeholder interviews. For instance, questions were asked on the expectations from the SCHT, and how their involvement in the accreditation system can help to improve FP services. Six stakeholders were interviewed in both rounds of data collection because they remained in positions of importance to the implementation of the accreditation system.

All stakeholder interviews were conducted by a consultant with postgraduate training in public health and extensive field experience, and who was not otherwise involved in the implementation of IntegratE’s activities. In preparation for data collection, the IntegratE project sent letters to all identified stakeholders and informed them that a consultant would be reaching out to them to conduct phone interviews. The consultant called each of the stakeholders to introduce herself, and to request for a convenient time to conduct the phone interview. In the first round of interviews, one person refused because she was not comfortable with a phone interview. In the second round, there were three refusals, each stating they were too busy.

In addition to the stakeholder interviews, there were two rounds of in-depth phone interviews with CPs and PPMVs from Lagos and Kaduna states, the program recipients of IntegratE’s pilot three-tier accreditation system. Participants were purposively selected, based on their involvement in the IntegratE project activities. Specifically, only those CPs and PPMVs who had attended an IntegratE-led training were eligible to be selected, and sampling in this group was random. In the first round of data collection, 11 CPs and 17 PPMVs were interviewed by phone between October and December 2020. In the second round, about 6 months later, 14 CPs and 16 PPMVs were interviewed by phone between May and June 2021. No program recipient was interviewed during both rounds of interviews, however, two and seven CPs refused to participate in the first and second rounds of data collection respectively. Program recipients were asked about their experiences with providing FP services in their respective communities; their experiences with referrals; knowledge and perception of three-tiered accreditation system; suggestions on improving the quality of FP services; relationship between CPs and PPMVs, and openness to a closer business relationship.

The consultant who was hired to conduct the stakeholder interviews held two half-day virtual training sessions with the four research assistants who conducted phone interviews among the program recipients. Data security, confidentiality, privacy, and the logistics of recording the interview are some concerns that other researchers have highlighted with virtual interviews [13, 14]. Thus, steps were taken before, during, and after training to ensure high quality interviews.

Before training, the hiring team ensured that only interviewers who had a good track record of conducting in-person interviews were invited for the study. Training included sessions on the uniqueness of phone interviews, particularly stressing the increased importance of ensuring privacy and confidentiality, as well as how to sense and respond to non-verbal cues [14, 15]. For instance, if an interviewer sensed that the respondent was distracted or in a hurry to finish the interview, they were trained to suggest that the interview be postponed to a more convenient time for the respondent. Furthermore, as recommended by other researchers, interviewers were taught to start by taking time to build rapport, then conduct the interview, and finally close on a friendly note [15]. Interviewers received feedback from the consultant trainer on the practice interviews they conducted as a training exercise, as well as one-on-one feedback on the first three interviews conducted during data collection. This feedback gave interviewers an opportunity to continue to improve on the quality of their interviews throughout the data collection process.

The choice of mobile phone rather than traditional face-to-face interviews was in order to reduce the risk of transmission of COVID-19 infection between participants and interviewers as a result of the pandemic. Additionally, the transition to phone interviews was in keeping with Population Council’s guidance at the time for data collection. Other researchers have reported the need for their teams to transition to virtual (e.g. video conferencing like Zoom; telephone) rather than traditional face-to-face interviews to minimize the risk of disease transmission and adhere to social distancing mandates [13–16].

Interviews were scheduled at a time that was most convenient for the participants, and using the language (English, Hausa, or Yoruba) preferred by the participants. For stakeholders, interviews were conducted by the Consultant, and interviewees were validated by making reference to previous interactions by email, phone and/or text. For selected program recipients, program staff from the IntegratE project informed them that interviewers would reach out to them to conduct a phone interview. Interviewers called potential respondents to fix a time for the interview, most frequently after having sent a text to introduce themselves. To ensure validity and confirm that the respondent was indeed the individual intended, interviewers asked questions to confirm their profession and the location of their pharmacy or drug shop. This information, including the phone numbers to call had been provided by IntegratE staff. All interviews were audio recorded, after obtaining informed consent and permission to turn on the audio tape. Poor connectivity was a frequent occurrence, but as has been previously documented, this experience was used to help to improve rapport between the interviewer and interviewee as they worked around this temporary setback [17]. Interviews with stakeholders and program recipients lasted 55 minutes, on average. For the stakeholders, since half of those interviewed in the second round were being interviewed a second time, fewer questions were asked and the interviews were much shorter on average (62 and 46 minutes on average in first and second rounds respectively).

### Data management and data analysis

Verbatim transcriptions, and translations to English were conducted. Verification of data integrity was conducted by having a team member who did not transcribe a given transcript to listen to the audio recording and make any necessary edits to the transcript. As part of this process, it was ensured that all transcripts were de-identified.

A thematic analysis of the data was conducted, following six steps previously suggested by Clarke and Braun [18, 19]. First, the consultant read through all the transcripts multiple times to become familiar with the data. Next, codes were identified and a codebook created. Codebook development was iterative, and involved three separate teams of three people, each independently reading the same set of 4–6 transcripts, coming up with their own codes, and discussing these as a team. The Consultant and two graduate students formed each team. Thereafter, the consultant assigned codes to segments of text across all the transcripts, using both deductive and inductive approaches. During this process, quotes that appeared well suited for a report or manuscript were identified and given an additional code, “finders’ keepers” for future reference.

In the third step, themes were constructed based on how the codes were related to each other. In the fourth step, themes were carefully reviewed, and sub-themes were created. In step 5, names were identified for themes and sub-themes based on their relationship to the overall study objectives. During the final step, representative quotes for each theme and sub-theme were identified and a subset, including some of those coded “finders’ keepers” were selected for the paper. To enhance credibility of the analysis process, the consultant met with 2–3 members of the IntegratE team on a weekly basis throughout data collection and data analysis to discuss and jointly interpret study findings. Data analysis was conducted using the computer-assisted qualitative software Atlas.ti (version 8.4.5). Data synthesis employed the use of memos, and reports generated in Atlas.ti.

## Results

In all, 15 stakeholders and 28 program recipients were interviewed in the first round of phone interviews, and 12 stakeholders and 30 program recipients in the second round.

The results of the analysis for this paper are presented in three themes that focus on: 1) the pilot three-tier accreditation system; 2) an enabling environment; and 3) implementation challenges. Below, we explore the responses from stakeholders and program recipients around these three themes and further discuss sub-themes for themes 1 and 2.

### Pilot three-tier accreditation system

Through the qualitative interviews, respondents discussed their impressions about the pilot three-tier accreditation system, namely, sharing their opinions about how the stratification of PPMVs is a game-changer, the significance of formal training for PPMVs, how shifting and sharing of FP tasks has helped to expand access to FP services, and the importance of establishing a functional referral system for patient care.

#### Stratification of PPMVs is a game-changer

Stakeholders were all familiar with PCN’s three-tier accreditation system, being piloted by IntegratE, and perceptions about the system were generally positive. They indicated that IntegratE’s piloting of the PCN three-tier accreditation system has helped to address shortages in human resources for health and provided additional FP training and skill acquisition for both CPs and PPMVs.

*“… if you compare our data of 2018 and 2019, you will see, you will really see the impact or the gap that these tiers have closed in terms of access to family planning services…”*

(Round 2; government official; Kaduna)

*“and with that we can reach a lot more, this is another cadre of health care providers [referring to PPMVs] that are available in rural area, hard to reach areas, in urban slum you see them everywhere, they are ubiquitous. And we need such cadre to be able to reach everyone in their reproductive age who deserve or desire to have family planning. But at the same time, we need to ensure that we have quality. So that’s where the tiered accreditation comes [in]…”*

(Round 1; federal government official)

Stakeholders perceived that the accreditation system helped to improve the status of PPMVs in their respective communities, and thus their self-confidence. There appeared to be assumptions that the three-tier accreditation system would transition from a pilot to a more permanent and sustainable addition to Nigeria’s health system. One government official felt that the accreditation system was so important that participation should not be a choice, but rather, *“… the tier accreditation should be mandatory.*” (Round 1; government official; Lagos)

#### Significance of formal training for PPMVs

The mandatory training of PPMVs using the standardized curriculum within SCHT was perceived by stakeholders as an essential piece of the three-tier accreditation model. Several stakeholders stated that using established schools for training was a good way to ensure acceptability of the model.

*“Well, well I think the Schools of Health Technology, they are in the best position to train them, if we want to really standardize the training of vendors [PPMVs]. They have the manpower, the requisite skills, teaching skills and what have you to carry out the training and then I think having the vendors [PPMVs] go through established schools for training is even… has even made it… made the training more authentic. So, I think the Schools of Health Technology are appropriate for the training of vendors.”*

(Round 2; government official; Lagos)

*“… it is a good thing to train them [PPMVs] because if you don’t know what you are doing, you can’t handle our people and it’s a kudos on the [Schools of] Health Technology that they are giving them this training…”*

Even though this stakeholder commended the training, she sounded a note of warning:

*“It’s not bad, we should train them, but we should be careful so that we’ll not go and hit our head with nails.”*

(Round 2; government official; Kaduna)

Stakeholders also provided information on the benefits and content of the training designed for PPMVs. Stakeholders were familiar with the content of the training to different degrees. Some knew first-hand what the scope of the training was while others had only limited second-hand knowledge. However, stakeholders appeared to be more informed about details of the formal training program during the second round of interviews, compared with the first round.

*“… the Pharmacist Council [is] trying to reposition them [PPMVs] to provide quality services because we need to reposition them. We can’t do away with them because in the rural areas, we don’t have adequate number of the professional healthcare service providers, so they fill in the gap. They are still relevant but they need to be properly trained…”*

[Round 1; federal government official]

*“So, the training has really you know, repositioned them and given them you know, the right path. ‘This is what you can do, this is what you cannot do,’ it has given them. They don’t… they don’t indulge… at least the ones I know they are not… they are more professional in what they do. They don’t indulge in selling under the counter so to say, that’s hide drugs to sell. They don’t indulge in that. They don’t go beyond what their license permits.”*

(Round 2; government official; Lagos)

*“I was saying what are the content of training and how rich are they? He [referring to someone she met at an event in a School of Health Technology] told me basically they were nothing much, but the emphasis was more on pharmacology and the way of handling medicine, that’s all.”*

(Round 2; government official; Kaduna)

*“… common ailments and diseases that are around, so we had trainings on immunization, asthma, hypertension, cholera, diarrhea, skin infections, diabetes, HIV & AIDS, family planning. Family planning was a little bit more elaborate… then we did effective communication… record keeping… pharmaceutical management, how to keep their shops… what they’re expected to do, what the program is all about… It was a quite comprehensive thing, I can’t, I’m not sure I covered all the topics but those are the few I can remember.”*

(Round 2; government official; Lagos)

To maintain and improve on the quality of training, stakeholders noted the need to introduce practical sessions and to organize retraining exercises for the teachers.

*“Well, they are doing their best… they are doing what they know how to do best: teaching. I just feel that the component, the training… there should be also a practical component of the training that will actually take the students to the field for practical sessions and what have you. That will also go a long way to improve… to add to their skills and knowledge. So, I think that, and then we can also retrain the trainers.”*

(Round 2; government official; Lagos)

#### Shifting and sharing tasks has helped to expand access to FP services

The pilot implementation efforts of IntegratE to train the CPs and PPMVs on FP service delivery has resulted in trained CPs and PPMVs who can now offer expanded FP services. To different degrees, many stakeholders expressed how the three-tier accreditation system helped to identify which tiers of PPMVs to shift and share certain FP tasks with, after training them to take on these responsibilities. A few stakeholders were so familiar with the accreditation system that they explained the specific training provided to each tier of PPMVs, noting that CPs received the same FP training as PPMVs in tiers 2 and 3. Many stakeholders perceived that this training has helped to make FP services more accessible to many more people, especially those who live in peri-urban, rural, and hard-to-reach areas of the two states. In the words of a government official:

“… *bringing that initiative of integrating PPMVs and CPs… was a welcomed, welcome decision and we have seen that it really, really improved our uptake and access*.”

(Round 1; government official; Lagos)

Program recipients also perceived that the tier accreditation has helped to improve access to family planning by making services more widely available:

“… *it [including CPs in delivery of FP services] has increased access to family planning for more people, that’s why I think it a good initiative… if they [PPMVs] are trained, properly trained. I believe the most important thing concerning family planning is for people to be able to access it.”*

(Round 1; CP; Kaduna)

*“In fact, after the coming of IntegratE, we see a lot of clients wanting to access the family planning services in our facilities… before, people, they [were] ashamed or they have fear about the family planning, but now, they came to realize it is a good option for them…”*

(Round 1; Tier 1 PPMV; Kaduna)

While embracing the opportunities that task shifting and task sharing have brought their way, PPMV leaders felt that more could be done by the IntegratE project to further expand their opportunities. For instance, one PPMV leader suggested that the private sector should be seen as “*partners in progress*” with the public sector, and that they [the private sector] should always have a seat at the table, that is, they should be involved in decision-making conversations. (Round 2; PPMV leader; Kaduna).

Another PPMV leader felt Tier 1 PPMVs should be allowed to do more:

*“… and then the other problem we have in this tier two, tier two were trained to train clients on how to give self Sayana injection... So, if I can tell a client, a patient or client to be injecting himself [herself], who doesn’t have any medical orientation, why can’t you train this tier one who has little, little medical background to be giving even if it is only Sayana injection?”*

(Round 1; PPMV leader; Kaduna)

Other PPMV leaders suggested that PPMVs could be more effective in their work if IntegratE assisted them to buy drugs and FP commodities from wholesalers at a cheaper rate, and advocate for the expansion of their essential drug list. A few of the PPMV leaders also suggested providing loans to PPMVs to help expand their business. In the words of one PPMV leader:

*“Task shifting, task sharing will be improved if the government will provide or make provision for soft loans to those private facility owners, the PPMVs, the community pharmacists, maybe a soft loan or free interest loan will be given to [PPMVs] at least to motivate them and to improve their business enterprises ...”*

(Round 2; PPMV leader; Kaduna)

Program recipients largely expressed similar perceptions and sentiments to their leaders. However, among the stakeholders, not everyone had a positive perception toward sharing FP service delivery and other health care delivery tasks with PPMVs. Some CP leaders and government officials were concerned that PPMVs were trying to take over the work of pharmacists, even in urban areas where PPMVs should not establish their shops. A couple of pharmacists shared their concerns as follows:

*“The universities will be producing more young pharmacists and then where will the pharmacists work?... If it’s the community, so if these patent vendors have saturated the ground… where will our own people, the pharmacists be, because we believe most pharmacists will come to the community to practice...”*

(Round 2; pharmacist & CP leader; Kaduna)

*“… why license PPMVs to be able to give injectables when the law is saying that they should not sell it, automatically you are giving them the license to sell injectables and then… what is the difference between them, by the time they start selling injectables, and the pharmacist? They are bringing them up to the same level with the pharmacist.”*

(Round 2; CP; Lagos)

It is expected that PPMVs will practice in rural and hard-to-reach areas, rather than in urban areas. It is generally believed that PPMVs should function where pharmacists are not available. Most pharmacists frown at PPMVs who open their patent medicine shops in urban areas. One community pharmacist summarized this turf war thus:

*“The reason why you can’t compare me [a CP]… with them [PPMVs], their, their own style of practice is different form my own style of practice and their license is not the same as mine… they are supposed to be in the rural areas but we are practicing in the urban areas with them… we can’t rub shoulders.”*

(Round 1; CP; Kaduna)

#### Establishing a functional referral system is important in patient care

Several stakeholders noted that all providers should understand that they have limitations, and a two-way referral system helps to improve the quality of the FP offered to clients by CPs and PPMVs. Apart from mentioning that the formal training program included discussions on referrals, only a couple of stakeholders explicitly stated that building capacity in referral of clients was an integral part of the three-tier accreditation system. References to referrals were largely in the context of offering safe and high quality health services by licensed providers with oversight by professional and regulatory bodies. Stakeholders discussed referrals from CPs and PPMVs to public health facilities. A few PPMVs were willing to refer to an appropriate CP near them while others felt doing so would mean losing clients for good.

CP and PPMV leaders, who themselves are providers, noted that referrals are largely given when the provider does not have or is not permitted to stock the drug/commodity (e.g. antibiotics or injectables for tier 1 PPMVs) or does not have the required skills (e.g. implant insertion/removal for CPs who had not been trained; management of adverse effects). In the words of a stakeholder:

*“In fact, even if they [FP clients] go to some facilities, not the community pharmacy… they will come if there is adverse effect or infection due to the method they used, maybe the small patent shops that are close, they [PPMVs] will now… refer them to us [CPs] so that we will now tell them [FP clients] the adverse effects of the medicine and give them advice on how to get better.”*

(Round 1; CP leader; Kaduna)

A functional referral system requires that providers understand what referrals entail, and they should be willing to provide care to anyone referred to them, regardless of the source of referral. However, one government official was concerned that some providers at higher level facilities may not understand the premise of referrals, judging by providers’ attitudes and actions. She said:

*“… I remember some CPs were complaining that even when they refer patients to the tertiary institutions or even the primary health care, most people look at it as,… they feel how can a CP be referring somebody to them [providers receiving the referral], they don’t seem to get what referral means... And I think the nurses, even doctors, they feel… that most times the patient comes back and they’ll just say what is their [client’s] business with a CP, that how can a CP be referring somebody to them [doctor/nurses], that the person should have come directly to them instead of going to a CP or a PPMV… So I think the people at the higher level, at the tertiary, primary health care, state and tertiary, I think they should be trained too, to understand what referral is and to understand why the CPs and PPMVs… to understand why patients are being referred to them and why they need to attend to the patient when the patient is being referred to them [by CPs and PPMVs]...”*

(Round 2, government official; Kaduna)

PPMV leaders also noted that clients reported that providers mistreat them because they were referred by a PPMV:

*“In coming back, one of the things they [clients] used to tell us is the way they [providers] attend to them, sometimes they’ll be shouting on them, sometimes… what we want them [providers] to do…, they will not achieve it, they [clients] will still come back… and give us feedback.”*

(Round 2; PPMV leader; Lagos)

### Enabling environment

The enabling environment theme reflects the perceptions of stakeholders regarding the supportive structures and processes required for full functionality and sustainability of the three-tier accreditation system. The sub-themes that emerged under the enabling environment theme included the need for improvements in licensing and supervision of CPs and PPMVs, the role of regulatory bodies, the advantage of including the private sector in FP service delivery, and the essence of supportive supervision.

#### Licensing and supervision of CPs and PPMVs need to be more effective

PCN is responsible for licensing and supervising CPs and PPMVs. One government official stressed the essence of the role of PCN in licensing as follows:

“*We want to be certain that whoever [referring to CPs and PPMVs] is practicing in the State has the license to practice because that is the only way in which that guarantees quality. Because everybody knows they have, they are to abide by rules and regulations like all other healthcare workers, and then you understand that when you’re rendering your service, they have to be done according to guidelines… that there are sanctions that can actually be placed on you… we don’t want to have quacks or people who think that they can get away with literarily damaging the health of our clients*.”

(Round 1; government official; Lagos)

CPs and PPMVs consistently suggested, directly or indirectly, that PCN needs to offer more support and provide opportunities to include CPs and PPMVs in trainings. A few respondents pointed to lapses in the regulatory roles of PCN as described below:

*“You know in this kind of country we are, they [PCN and Ministry of Health] will just support you verbally. (Laughter)… They will just come and see how the place is, they will encourage you, if you are doing well, that is the only support they give us… Apart from that, I don’t see anything, they don’t give us any support, in finance or even training. You know, as they’re part of this, they are supposed to be inviting us for training…. If not because of IntegratE… I could have not start the family planning service.”*

(Round 1; Tier 2 PPMV; Kaduna)

*“They [PCN] only have to improve in our area, now, I think I’m the only person who is accredited and registered with PCN… [For] the rest of them [unregistered patent medicine shop owners], they [PCN] have never come and check or talk to them [unregistered shop owners], or close their shops, so, we have been telling them [PCN] that, we [those who are registered] have people around us, who are not even health workers… But they [unregistered patent medicine shop owners] are selling drugs and no any action has been taken… So, these things discourage us [those who are registered].*”

(Round 1; Tier 2 PPMV; Kaduna)

#### Regulatory bodies have an important role to play in ensuring quality health services

While acknowledging the usefulness of including PPMVs in the quest to expand access to FP services after adequate training, several stakeholders sounded a note of caution, suggesting that regulation of PPMVs should be fully implemented to keep them in check. Several program recipients, particularly CPs echoed this sentiment, as revealed in the following quote:

*“It’s a good one; only if they [PCN] will be able to supervise and follow up… Not that you’ll just train them [PPMVs] and leave them to be on their own… They [PCN] have to, I mean regularly, monitor what they are doing.”*

(Round 1; CP; Lagos)

One CP in Lagos state was particularly critical of giving PPMVs more responsibilities, noting that they already go beyond the scope of their practice:

*“So, do you know that so many of those things that we in the pharmacies don’t do, all these PPMVs they do it, so they will always go beyond the boundary.”*

(Round 1; CP; Lagos)

While the CP and PPMV leaders said they themselves had not been harassed by regulatory bodies, a few noted that they were aware of other CPs or PPMVs that had. The consensus appeared to be that those who were harassed either were not licensed or they engaged in nefarious activities. The program recipients corroborated this perception. One PPMV had this to say:

*“Most especially when you have the qualification that you can stand on, nobody can harass you, even if they [PCN] come and you show them your license or something relevant to that area, they [PCN] will not harass you, except if you don’t know what you are doing or you go beyond what you are supposed to do…”*

(Round 1; Tier 2 PPMV; Kaduna)

#### The private sector advantage

Some of the advantages of bringing the private sector into service delivery programs for FP include the geographically shorter distances to travel for services, minimal waiting times when seeking care (time savings), and services that are private, discreet and personable. Moreover, the geographical distribution of PPMVs has helped to ensure the ubiquity of basic health and FP services, particularly in rural areas, urban slums, and hard-to-reach areas. One PPMV leader described the ubiquity of PPMVs in the following way:

*“We even have a slogan where we say, where there is no community, there’s no NAPPMED [National Association of Patent and Proprietary Medicine Vendors], where there’s no NAPPMED, that’s the name of our association, where there is no NAPPMED, there is no community. So anywhere you have a community, there must be our member there, and our member is there to serve the need of the people in that community and government cannot provide health facilities in all the communities nationwide.”*

(Round 2; PPMV leader; Kaduna)

A government official from Kaduna described how access to FP services is easier for community members, since CPs and PPMVs are now offering the services:

*“… because most of the people in the community, their first point of call is the PPMVs and the CPs. Now that this family planning, access to family planning has been brought down to, to the grassroot, to the CPs and PPMVs… People have access, it’s easier for people to access family planning, to have access to family planning than it was before…”*

(Round 2, government official; Kaduna)

Program recipients also indicated that clients or patients come to them before going to hospitals:

*“… we [community pharmacists] are the first point of call people meet before going to the hospital.”*

(Round 2, CP; Lagos)

*“We [community pharmacists] also render family planning services at our level to serve the community and this is the first point of call for all clients.”*

(Round 2, CP; Kaduna)

#### Integrated supportive supervision

Across stakeholder groups, the consensus was that supportive supervision was an important piece of the enabling environment that helps to assure the delivery of high-quality FP services by PPMVs trained as part of the three-tier accreditation system. Training, mentoring, monitoring and supervision were perceived to be important components of ensuring quality FP services. Both stakeholders and program recipients noted that supportive supervision was essential in correcting wrong practices within the workplace, and that the visits were not meant to be fault-finding, but to reflect efforts at improving the skills of the provider and the services offered to clients. Integrated Supportive Supervision Visits (ISSVs)^1^ were viewed positively and personal experiences shared by stakeholders who were either supervisory team members or representatives of associations were appreciative of the process and believed that the individualized visits helped to improve quality of FP services. Some PPMV leaders and a government official suggested greater involvement of the associations at the state level in routine supportive supervisory visits as a way of addressing some of the capacity limitations of PCN, and improving the quality of services offered.

*“When they [leaders of associations] see that [the] government engage[s] with them [associations] and government sees their relevance, they [leaders of associations] want to do a lot to ensure that government policies, government programs which their members are not really respected but fully enforced. So, I feel Pharmacist Council should engage even more deeply in their professional association that, that are affiliated to them.”*

(Round 1; government official; Abuja)

Most program recipients interviewed felt that the supportive supervision they received was effective. Participants provided insights on the importance and usefulness of the one-on-one guidance they received, particularly from the IntegratE project, as well as from the ISSVs:

*“And it’s very effective because if you are lazy and you have been failing, they will correct you and talk one-on-one and if you have any challenge, you talk to them.”*

(Round 1; Tier 2 PPMV; Kaduna)

A PPMV from Kaduna state discussed how the supervisory visits helped him to make structural improvements to his shop and to arrange things properly:

*“You know sometimes, we just do the business [referring to running a patent medicine shop] to get money. I have improved on how to keep my shop very clean, that’s one. I have also improved on how to have a cross ventilation in my shop, that no matter how the shop is, there should be a small window, that when I close the shop at night, air can still penetrate… And also, how to arrange things properly, where to keep the waste, used syringes and used swabs differently, but before, we don’t do that.”*

(Round 1; Tier 2 PPMV; Kaduna)

Some stakeholders, specifically PPMV leaders and government officials noted that ISSVs built confidence in the quality of services offered by PPMVs. The following quote from a PPMV leader demonstrates how the supportive supervision visits provide instruction to improve service delivery:

*“… this supportive supervision really has seen to make sure that quality family planning is given by the providers. Because in any of the supportive supervision… in any shop that we’ve attended, the provider will be asked to demonstrate how she normally receive(s) a client when they come to the shop, how she counsel(s) them… they do that so if there’s any mistake from the provider, the supportive supervision group will correct you and tell them the right thing to do. Anywhere there’s any lapses, they will correct him and they will make sure they check the register, that everything are recorded accordingly. If there is any mistake in the recording, the provider will be corrected. If there is correction in the counselling, he is being corrected. Anything that needs to be corrected used to be corrected and you know as human beings, you train a person after one, two, three, four, five months, some normally they will relax, they may forget some of the things that you teach them… So, if there is any mistake at any level then after everything, you come back and correct the provider, the provider on what he is supposed to do and after correcting him, you will still ask him again to know… whether he has taken the correction.”*

(Round 2; PPMV leader; Kaduna)

One government official, while praising the supervisory visits and referring to her experience being involved in them also indicated that the visits were useful to keep PPMVs in check, so that they do not go outside the scope that had been assigned to them as part of the accreditation system:

*“Yes, I’m there on the field with them, I see what they do and sometimes I’m really impressed… and the reason they’re able to provide these services is because they were trained, although to be sincere with you, some of them go outside their scope and that is why the supervisory visit is important. When we go out, we see those going outside their scope… we tell them what to do and then when we go for our own normal inspections as Pharmacist Council of Nigeria, we issue compliance directives to them so that they don’t go beyond their scope. But really, the ISS visits have been of great help.”*

(Round 2; government official; Kaduna)

### Implementation challenges

While most stakeholders felt stratification of PPMVs was a welcome development, some concerns were raised. Interviews with various stakeholders revealed that there was deep-seated mistrust between CPs and PPMVs as they often saw themselves as rivals and competitors. Several stakeholders expressed the need to ensure widespread sensitization of both stakeholders and program recipients, noting that this was an essential step in addressing whatever implementation challenges existed, as expressed by a government officer in Lagos as follows:

“*… we really need to do a bit more sensitization, get them [CPs and PPMVs] together so that we see the reasons why these things must be done… yes, I also feel that the vendors [PPMVs] too should also be enlightened, educated… As to, as to know their limits, yes to know, know their limits, not to go beyond your [their] limits. If you are in tier one, still remain at tier one… Don’t go, don’t go doing what you are not licensed or you have not been trained to do*.”

(Round 1; government official; Lagos)

Several other stakeholders emphasized the need to bring stakeholder groups together in order to address some of the implementation challenges. For instance:

*“It should be leaders of all the stakeholders to discuss the matter: the PCN, the leaders of the ACPN and the family planning stakeholders and the leaders of the PPMVs to discuss the matter, inviting the president [of the associations]… As we’re doing this interview now, a stakeholders’ meeting where we can look at each other’s eyeballs and discuss [the] way forward.”*

(Round 2; PPMV leader; Lagos)

A CP in Kaduna reported that his colleagues told him that PPMVs tend to overstep their limits regarding what they are allowed to stock:

*“I learned from my colleagues… some of the Patent Medicine Vendors they tend to go beyond what they are supposed to stock… so that one is a challenge; but from my own part, in my own area I don’t have any patent medicine vendor near me.”*

(Round 1; CP; Kaduna)

Almost every stakeholder spoke with deep concern about the delays in licensing PPMVs as a potential implementation setback that could threaten the success of the three-tier accreditation system. This point was corroborated by the program recipients who were interviewed as well. The PPMVs spoke of their personal frustrations and experiences in trying to get licensed. Both stakeholders and program recipients noted that only a small fraction of those practicing as PPMVs were licensed. This implies that the requirement to be licensed in order to benefit from IntegratE’s supported pilot three-tier accreditation system already excluded most of those who were practicing as PPMVs in Lagos and Kaduna states.

Other challenges highlighted included inadequate staff for site inspection and supervisory visits, and an insufficient number of vehicles for site inspection and quarterly ISSVs. CP and PPMV leaders also noted that the regulatory bodies hardly ever visited the premises of CPs and PPMVs after the initial location inspection required for their license. There were suggestions to make the licensing process easier and smoother, but both stakeholders and program recipients were aware of the challenges faced by the licensing authority, PCN that made their suggestions difficult to implement. One PPMV leader had the following suggestion:

*“I think the PCN have to employ more… they have to employ more people to do the job for them… Considering the number of PPMVs across the state, the PCN has to employ more… more officials...”*

(Round 2; PPMV leader; Tier 1; Kaduna)

## Discussion

This paper shares perceptions of different groups of stakeholders regarding the pilot three-tier accreditation system for PPMVs in Lagos and Kaduna States of Nigeria and draws on the WHO guidelines for TS in describing IntegratE’s activities. The perceptions and experiences of various stakeholders regarding the pilot three-tier accreditation system and the environment that enables its full implementation are varied, but largely positive. Stakeholders acknowledged that the pilot three-tier accreditation system is an innovative approach. The system recognizes and taps into the ubiquity of PPMVs in otherwise hard-to-reach and rural areas. Moreover, the system is beneficial, both to clients and private sector providers. For clients, this accreditation system provides opportunities for closer physical access to services, more personable interactions with providers, and less time lost traveling long distances to obtain care. CPs and PPMVs, on the other hand gain from the improved social standing, more respect in their practice communities, new skills, and more confidence. This accreditation system was perceived to have expanded access to FP by bringing high quality FP services closer to people where they live and work, a finding similar to that reported in the ADDO program [8, 9].

Even though six of the 15 stakeholders interviewed in the round 1 interviews were also interviewed in round 2, there is no evidence to show that the change in participants affected the findings reported on perceptions of the three-tier accreditation system. This may be because the stakeholders who were interviewed in one rather than in both rounds were also involved in the implementation of the three-tier accreditation system. Typically, when the Director/Chairperson/Coordinator was not available, the Deputy spoke with the Consultant, or vice-versa. The exception to this is the variation in responses due to the actual roll out of the MEPTP through the Schools of Health Technology between the first and second rounds of interviews. Respondents had a better understanding of what this component of the pilot accreditation system was about, and were more confident in speaking about it. Some were directly involved as facilitators or program recipients, and thus spoke from experience. The sample of program recipients was completely different in the two rounds of data collection, but their perceptions of the accreditation system were similar.

Continual engagement with the private sector can help to further expand FP services in Lagos and Kaduna states, but the IntegratE project will need to put measures in place to ensure that PCN, ACPN, PPMV associations, and other organizations continue to sustain the wins that have been observed. For instance, PPMVs, especially those in Tier 1, are reported to often overstep their boundaries, and thus require improved supervision by PCN, the regulatory body. Other researchers have documented the poor regulation of PPMVs in Nigeria, and associated safety concerns [20]. For the three-tier accreditation system to thrive, an enabling environment must be provided by PCN, the regulatory body, as well as continued supportive supervision by an integrated team for continuous improvements in service delivery. The integrated supportive supervision team should include representatives from all stakeholders - the State MOH, State Primary Health Care Board, PCN, ACPN, and PPMV Associations. This collaboration will ensure optimal regulation and supervision of CPs and PPMVs and should help to reap the benefits of public-private partnerships in healthcare delivery, but must be properly funded [2, 9].

Apart from supportive supervision, a well laid out, functional two-way referral system can help to provide the right enabling environment, and improve services offered to clients and patients. However, the findings of this study suggest that concerted efforts must be made to ensure providers understand how referrals can create a win-win situation, benefitting both the client and the provider. This study, like other similar qualitative research suggests the use of referrals may be sub-optimal [21] without sufficient sensitization and acceptance from higher level providers to receive referrals from CPs and PPMVs. Unfortunately, while development of the capacity of PPMVs for referrals is an integral part of the three-tier accreditation system, there is nothing in place to ensure that health workers in facilities in the public sector are willing and prepared to receive and manage referred clients and patients in a respectful manner. The implication of this is that a one-way referral, rather than a two-way referral is inadvertently being promoted. For clients and patients to receive high quality care at all levels, the public and private sectors must work hand-in-hand to create and maintain one health system.

The mandatory training component of the three-tier accreditation system has been enthusiastically received. However, a major critique of this component of the accreditation system is that trainees will have to pay out-of-pocket to participate in the mandatory training after the donors pull out. While the new knowledge and skills benefit the PPMVs, and by extension their clients and patients, some of them may find it difficult to personally fund their participation at a 4-week training. The costs go beyond the fees for the training, as they will need to find ways to keep their shops open so they can continue to make profits, even while they are away. It is imperative for PCN to work with the PPMV association leaders to brainstorm on potential ways to ensure that participation at the mandatory training does not constitute a huge financial burden to individual PPMVs, and thus a deterrent to the full accreditation of these providers and their shops. If not given the priority and full attention it deserves, this financial hurdle can potentially pose a huge threat to the sustainability of the accreditation system.

Another challenge that the stakeholders in this study identified is the slow licensing process for PPMVs and delayed site inspection for patent medicine shops, with little or no enforcement on licensing of patent medicine shops. This challenge reveals a need to shorten processing time and strictly enforce licensing of patent medicine shops. Only a fraction of PPMVs who practice are licensed to do so [6]. Inadequate funding for the needed manpower, transportation, and general running costs is probably why the licensing process is so slow. The sustainability of the system is at stake unless clear locally generated funding sources are identified and put in place to support site inspection, an integral part of the system. In this technological age, the licensing process should be fully automated and online, shortening the time taken, and reducing the needed manpower. Site visits can also be automatically scheduled using available software packages to link inspection teams to the patent medicine shops and community pharmacies they need to visit to accredit the premises. Such automated processes will free staff time to participate in other important activities, such as ISSVs. In an update from the Registrar of PCN, he noted that the Council is working on automation. In his words, “In line with the ongoing automation project going on at the Council, it is important to note that Patent and Proprietary Medicine Vendors License (PPMVL) registration is now fully online. A number of processes and examinations have already been earmarked for automation this year” [22].

Although the pilot three-tier accreditation system has made some noticeable gains, there is a need to manage expectations to be able to build on these gains. For instance, some PPMVs feel they should be allowed to do more, such as have an expanded list of drugs they can sell, and give Sayana Press (Depo medroxyprogesterone acetate-subcutaneous injection). These perceptions should be addressed during open conversations with the professional associations, in order to manage expectations, and ensure harmony. Frequent communication through meetings and open conversations have been described as opportunities to build trust and improve team harmony in healthcare settings [23–25].

The overall positive perceptions of stakeholders and program recipients interviewed is a limitation of this study, as the views presented may not be representative of all stakeholders and all program recipients in Kaduna and Lagos states. To encourage openness, all interviews were conducted by consultants who were not otherwise involved in the implementation of the accreditation system, and were not on the payroll of PCN or the IntegratE project. Given the Nigerian culture of appreciation of programs seen as beneficial, we constantly encouraged participants to speak freely and reminded them that the information they shared would be kept confidential. We let them know that their names would not be associated with the audio tape or transcript from the interview, and that all data would only be shared in the aggregate. Apart from while obtaining informed consent, these messages were repeated as frequently as necessary during the course of the interview, especially when it appeared the participant was holding back thoughts or information. In view of the foregoing, it is important to exercise caution when interpreting the findings of this study for other settings.

The implementation research endeavor described in this paper has implications for programs, policy and further research. Programs like IntegratE can support desired improvements in services by playing the role of a neutral, cohesive body that brings all stakeholders together with the government to address their differences and work together to attain a common goal, such as improving access to FP services. The TS policy is currently being revised, based on field experience, and should clearly articulate sanctions for PPMVs who go beyond their scope of practice. The policy will need to be domesticated by individual states, as their buy-in is essential to the success of task shifting. In anticipation of the revised TS policy, PCN and states where PCN is working gave waivers to enable PPMVs (Tiers 2 and 3) and CPs to be trained and to be able to offer injectables and implants. The federal and state governments will need to allocate funds to ensure that the revised TS policy is fully implemented.

The Federal Government of Nigeria can support TS by ensuring the prompt completion and dissemination of the revised TS policy. There should be a costed implementation plan for TS that will include budgets for training and supportive supervision to enhance the quality of service delivery. Pending the release of the revised policy, the Federal Government should continue to provide waivers to state governments to allow trained PPMVs to administer a broader range of FP commodities. The state and local governments can domesticate the TS policy within their jurisdiction. Governments at all levels can support the private sector, including PPMVs by linking them to FP commodities sold at control prices. More research is needed to better quantify and understand the increase in FP access and improvement in quality of services offered as a result of TS family planning services to CPs and PPMVs. Such information can be used to further advocate for funds to expand IntegratE’s work in Nigeria.

## Conclusion

Stakeholders based in Kaduna and Lagos States had largely positive perceptions of Pharmacists Council of Nigeria’s pilot three-tier accreditation system for Patent and Proprietary Medicine Vendors to expand access to family planning services. Program recipients also expressed their appreciation for the three-tier accreditation system, and the opportunity to gain new skills and offer additional services to their respective communities. Ensuring an enabling regulatory and supervisory environment is critical to the success of this system. Although there are a few challenges that need to be addressed, scaling up this three-tier accreditation system has the potential to expand access to FP services in Nigeria. Frequent communication and open conversations between all stakeholder and program recipient groups and widespread dissemination of an inclusive, revised task shifting policy document are recommended. IntegratE will continue to work with all relevant stakeholders to improve on the gains so far, and scale up the tier accreditation system to other Nigerian States.

## Supplementary Information

**Additional file 1.** Interview Guide for Stakeholders in Kaduna and Lagos States at Baseline and Follow-Up.

**Additional file 2.** Interview Guides for Community Pharmacists and Patent and Proprietary Medicine Vendors in Kaduna and Lagos States at Baseline and Follow-Up.

## Data Availability

The de-identified dataset analyzed during the current study will be available on the Population Council’s Dataverse, https://dataverse.harvard.edu/dataverse/popcouncil. The data will also be available from the corresponding author on reasonable request.

## Abbreviations

ACPN: Association of Community Pharmacists of Nigeria
ADDO: Accredited Drug Dispensing Outlet
CP: Community Pharmacist
FP: Family planning
ISSVs: Integrated Supportive Supervision Visits
MEPTP: Mandatory Entry Point Training Program
MOH: Ministry of Health
NAPPMED: National Association of Patent and Proprietary Medicine Vendors
PCN: Pharmacists’ Council of Nigeria
PPMV: Patent and Proprietary Medicine Vendors
SCHT: Schools and Colleges of Health Technology
TS: Task shifting
WHO: World Health Organization

## Footnotes

1 Integrated Supportive Supervision Visits, ISSV refer to visits by the joint team consisting of representatives from the SMOH, PCN, Primary Health Care Board, ACPN, who visit IntegratE-trained pharmacists and PPMVs to assess FP service provision and give onsite support on identified gaps, using a checklist. The ISSVs are part of the project sustainability plan to make sure supportive supervision of CPs & PPMVs is integrated into government SSV, even after the project ends.
