# Supplementary material for: Nigerian stakeholders’ perceptions of a pilot tier accreditation system for Patent and Proprietary Medicine Vendors to expand access to family planning services
Source: BMC Health Serv Res. 2022 Sep 3;22:1119. doi: 10.1186/s12913-022-08503-3 (PMC9440463; doi:10.1186/s12913-022-08503-3)
Supplement: Supplementary file 1 — Additional file 1. Interview Guide for Stakeholders in Kaduna and Lagos States at Baseline and Follow-Up. [file 12913_2022_8503_MOESM1_ESM.pdf]

Supplementary File 1. Interview Guide for Stakeholders in Kaduna and Lagos States at Baseline and Follow-Up

**Baseline Interview Guide: Stakeholders**	**Follow Up Interview Guide: Stakeholders**
**Respondent ID: [__|__|__|__|__|__]**<br><br>**Date:**<br><br>**INTRODUCTION**<br>1. To start, could you tell me a little bit about yourself and your work related to family planning and/or primary health care?<br>a. Probe: What influenced your decision to pursue your current line of work?	**Respondent ID: [__|__|__|__|__|__]**<br><br>**Date:**<br><br>**INTRODUCTION**<br>1. To start, could you tell me a little bit about your ongoing work related to family planning and/or primary health care?
2. In your official role, how do you interact with CPs and PPMVs?<br>a. *Follow-up*: In what other ways have you interacted with CPs and PPMVs in the past?<br>i. Official interactions?<br>ii. Non-official or personal interactions?	2. In your official role, how have you interacted with CPs and PPMVs over the past 6 months?<br>a. *Follow-up*: In what other ways have you interacted with CPs and PPMVs over the past 6 months?<br>b. *Probe*: Please share your personal experience with seeking services from CPs and PPMVs over the past 6 months?
3. What are your thoughts on Nigeria's task shifting and task sharing policy as it relates to expanding access to family planning and primary health care services?	**TASK SHIFTING, TASK SHARING, AND SUPPORTIVE SUPERVISION**<br>3. In your opinion, how has Nigeria faired in rolling out her task shifting and task sharing policy as it relates to expanding access to family planning? How about with regard to expanding access to primary health care services?<br>a. *Probe:* Please share your opinions on improving task

	shifting and task sharing in Nigeria.<br>b. *Follow up:* How can task shifting and task sharing be improved in the private sector?<br>c. *Follow up:* What changes would you like to see in the implementation of the task shifting and task sharing policy in the public sector?
	4. IntegratE instituted Integrated Supportive Supervision (ISS) to improve the quality of FP service delivery and monitoring by the SMOH. Have the ISS efforts helped to build your confidence in the quality of FP services offered by PPMVs? Why or why not? Please explain.
4. What experiences do you have with supportive supervision? Any others?	5. What experiences have you had with supportive supervision for CPs and PPMVs over the past 6 months? Any others?
	**EXPECTATIONS FROM THE SCHOOLS OF HEALTH TECHNOLOGY**<br><br>INT: Thank you for sharing this information with me. I would like to ask you a few questions related to your expectations regarding the role of the Schools of Health Technology.
	6. PPMVs, classified into different tiers based on whether or not they have health qualifications, are now being trained by PCN through the Schools of Health Technology. Are you aware of this development?<br>a. *Follow-up*: What are your thoughts on the current role of the Schools of Health

<table>
<tr><td></td><td>Technology in the training of PPMVs, based on the tier they have been assigned to?</td></tr>
<tr><td></td><td>7. What suggestions do you have regarding how the involvement of the Schools of Health Technology can better improve the quality of family planning services in Nigeria?</td></tr>
<tr><td>THREE-TIER ACCREDITATION SYSTEM

INT: Great, thank you for sharing with me.
5. How are CPs currently licensed and supervised? How are PPMVs currently licensed and supervised?
a. Probe: Ask for a detailed description of the licensing procedure, if known.
b. Probe: Ask for supervisory services provided: Training, monitoring visits, data management.
c. Follow up: How effective are these licensing and supervisory systems?
d. Follow up: What is your level of involvement in the licensing and/or supervision of CPs or PPMVs?
e. Probe: Would you like to be more involved? Why or why not?</td><td>THREE-TIER ACCREDITATION SYSTEM

INT: Great, thank you for sharing with me. Now I’d like to speak with you about the three-tier accreditation system.</td></tr>
<tr><td></td><td>8. What do you know about PCN’s Mandatory Entry Point Training Program (MEPTP) also called the Tier Accreditation Training for PPMVs?
a. Probe: Ask for where it has started, if known (specific Schools of Health Technology).</td></tr>
</table>

	b. *Probe:* Ask for **when** it started, if known.<br>c. *Probe:* Ask if respondent knows that some PPMVs have already been trained. If yes, ask what stakeholder has heard about the training since it started.<br>d. *Probe:* What is the content of the training?<br>e. *Probe:* How long does the training last?<br>f. *Follow up:* Are you a master trainer or involved in the MEPTP in any other way?
I would now like to ask you about your perception of, and experiences with IntegratE's three-tier accreditation system.<br><br>6. Have you heard about PCN/IntegratE's three-tier accreditation system? Please elaborate.<br>(As you know) In this system, PPMVs are grouped into 3 tiers: non-health qualified PPMVs, health qualified PPMVs, and pharmacy technicians, and are allowed to perform specific family planning tasks based on the tier they belong to.	
7. How do you perceive PCN/IntegratE's three-tier accreditation system?<br>a. *Probe:* How do you think this system improves on what obtained prior to 2018 when CPs and PPMVs who offered specific family planning services did not have any formal training or support to do so?	

8. What effects, if any, do you think the three-tier accreditation system may have on family planning services?	
9. What implementation challenges do you see with the three-tier accreditation system?<br>a. *Follow up:* How can these challenges be overcome?<br>b. *Follow up:* What are your recommendations for improving the three-tier accreditation system?<br>*c. Probe:* Any other challenges? *If not mentioned, specifically ask for challenges related to implementation cost and securing sufficient political will.*	9. What implementation challenges do you see with the three-tier accreditation system that allows PPMVs to provide PHC and FP services?<br>a. *Follow up:* How can these challenges be overcome?<br>b. *Follow up:* What are your recommendations for improving the three-tier accreditation system?<br>*c. Probe:* Any other challenges? If not mentioned, specifically ask for challenges related to implementation cost and securing sufficient political will.
**EXPECTATIONS FROM THE ACCREDITATION SYSTEM**<br><br>INT: Thank you for sharing this information with me. I would like to ask you a few questions related to your expectations regarding the PCN accreditation system.	
10. What do you know about the proposed accreditation system after the end of IntegratE? (Where PPMVs will be trained by PCN through the School of Health Technology and classified into different tiers based on whether or not they have health qualifications)<br>*a. Probe: Perceived impact on business* (the number of clients, clients' and the community's confidence in	

services offered, harassment from regulatory and police agencies).<br>b. *Follow up:* What are your thoughts on incorporating the School of Health Technology into the tier accreditation system?	
11. What suggestions do you have about how the PCN accreditation system can better improve the quality of family planning services in Nigeria?	
**HUB AND SPOKE SUPERVISION MODEL**<br><br>INT: Thank you so much. I would now like to ask you about your perception of, and experiences with IntegratE's hub-and-spoke supervision model (where a community pharmacist is supervising/supporting a group of PPMVs on pharmaceutical care and drug management).	**HUB AND SPOKE SUPERVISION MODEL (For stakeholders from PCN only)**<br><br>INT: Thank you so much. I would now like to ask you about your perception of implementation challenges with IntegratE's proposed hub-and-spoke supervision model (where a community pharmacist will supervise/support a group of PPMVs on pharmaceutical care and drug management).
12. How do you perceive PCN/IntegratE's hub and spoke supervisory model?<br>a. *Probe*: How do you think this supportive supervisory system improves on what obtained prior to 2018 where PCN was only focused on enforcement of and compliance with policies and laws related to drug stocking?	10. How do you perceive PCN/IntegratE's hub-and-spoke supervisory model?<br>a. Please share any benefits you think the model has.<br>i. What beneficial effect, if any, can the model have on quality of services offered by PPMVs?<br>ii. What beneficial effect, if any, can the model have on appropriate use of referral services?<br>iii. Any other potential benefits of the model?

13. What effects, if any, do you think the hub and spoke supervisory model may have on drug stock practices of PPMVs?<br>a. *Probe:* Can this model improve drug stock practices of PPMVs? Why or why not?	
14. What effects, if any, do you think the hub and spoke supervisory model may have on stocking of family planning commodities?<br>a. *Probe:* Do you think this model can help to avoid stockouts of commodities? Why or why not?<br>b. *Probe:* Do you think this model can help to avoid stocking of expired drugs? Why or why not?	
15. What implementation challenges do you see with the hub-and-spoke supervisory model?<br>a. Follow up: How can these challenges be overcome?<br>b. Follow up: What are your recommendations for improving the hub and spoke supervision model?	11. What implementation challenges do you see with the hub-and-spoke supervisory model?<br>a. *Follow up*: How can these challenges be overcome?<br>b. *Follow up*: What are your recommendations for improving the hub-and-spoke supervision model?
**Closing**<br><br>Thank you very much for you time and the thoughtful information you have shared with me today!<br><br>Do you have any final comments before we end our discussion?	**Closing**<br><br>Thank you very much for your time and the thoughtful information you have shared with me today!<br><br>Do you have any final comments before we end our discussion?
