# Supplementary material for: Nigerian stakeholders’ perceptions of a pilot tier accreditation system for Patent and Proprietary Medicine Vendors to expand access to family planning services
Source: BMC Health Serv Res. 2022 Sep 3;22:1119. doi: 10.1186/s12913-022-08503-3 (PMC9440463; doi:10.1186/s12913-022-08503-3)
Supplement: Supplementary file 2 — Additional file 2. Interview Guides for Community Pharmacists and Patent and Proprietary Medicine Vendors in Kaduna and Lagos States at Baseline and Follow-Up. [file 12913_2022_8503_MOESM2_ESM.pdf]

Supplementary File 2. Interview Guides for Community Pharmacists and Patent and Proprietary Medicine Vendors (below) in Kaduna and Lagos States at Baseline and Follow-Up

<table>
<tr><td>IntegratE</td><td>IntegratE</td></tr>
<tr><th>Interview Guide:
Community Pharmacists</th><th>Follow Up Interview Guide:
Community Pharmacists</th></tr>
<tr><td>Respondent ID: [__|__|__|__|__|__]

Date:

INTRODUCTION
1. To start, could you tell me a little bit about yourself and your job as a community pharmacist? What influenced your decision to become a CP?</td><td>Respondent ID: [__|__|__|__|__|__]

Date:

INTRODUCTION
1. To start, could you tell me a little bit about your work as a community pharmacist in Lagos/Kaduna state?
a. Follow-up: When did you enroll with the IntegratE project, and why?
b. Follow-up: How long have you been providing family planning services?</td></tr>
<tr><td>CURRENT PRACTICE WITH FAMILY PLANNING

INT: Great, thank you for sharing that with me. I would now like to ask you about your experiences with providing family planning services in your pharmacy.

2. Can you describe your experience with providing family planning services (products and counselling) in your community?
a. Probe: In your opinion, what do clients think about the family planning services you provide (women versus men)?
b. Follow-up: What challenges, if any, have you experienced in providing family planning services in your community? Please explain.</td><td>CURRENT PRACTICE WITH FAMILY PLANNING

INT: Great, thank you for sharing that with me. I would now like to ask you about your experiences with providing family planning services in your pharmacy.

2. Can you describe your experience with providing family planning services (products and counselling) in your community?
a. Probe: In your opinion, what do clients think about the family planning services you provide (women versus men)?
b. Follow-up: What challenges, if any, have you experienced in providing family planning services in your community? Please explain.</td></tr>
</table>

i. *Probe:* Specific challenges – cultural, religious, poor client patronage, regulatory, etc.<br>ii. *Preferences:* What have you observed regarding client preferences for specific family planning services? Please elaborate.<br>iii. Are there other methods you wish you could sell or provide? (if yes, please explain)	i. *Probe:* Specific challenges – cultural, religious, poor client patronage, regulatory, etc.<br>ii. *Preferences:* What have you observed regarding client preferences for specific family planning services? Please elaborate.<br>iii. Are there other methods you wish you could sell or provide? (If yes, please explain.)<br>c. *Follow-up*: Beyond family planning services, can you describe your experience with providing primary health care services in your community?<br>i. Are there any other services you provide in your pharmacy? Please explain.
3. In what situations would you refer a client to a health facility or another CP?<br>a. *Follow-up:* What factors facilitate referrals for family planning methods?<br>b. *Follow-up:* What do you find difficult about referring clients to a health facility or another CP for family planning services?<br>c. *Follow up:* Do you receive feedback from the facility you refer clients to, or from the clients themselves? Please explain.	3. In what situations would you refer a client to a health facility or another CP?<br>a. *Follow-up:* What factors facilitate referrals for family planning methods?<br>b. *Follow-up:* What do you find difficult about referring clients to a health facility or another CP for family planning services?<br>c. *Follow up:* Do you receive feedback from the facility you refer clients to, or from the clients themselves? Please explain.
**FAMILY PLANNING TRAINING AND REPORTING**<br>4. Have you been invited to any training on FP organized by IntegratE? Organized by another organization? (*Please ask for name of organization*.)<br>a. *Follow up:* Did you attend the training? How valuable was the	**FAMILY PLANNING TRAINING AND REPORTING**<br>4. Have you been invited to any training on FP organized by IntegratE? Organized by another organization? (*Please ask for name of organization*.)<br>a. *Probe:* When did you attend the training?

training to your practice? Please explain.<br>b. *Follow up:* Have you been reporting your service statistics monthly as requested at the training?<br>i. **If yes**, "Have you experienced any barriers to regular reporting?"<br>1. *Probe:* Lack of time to document FP service into the register and monthly summary form? Not comfortable with the DHIS reporting platform? Lack of clients to report on? Challenge with internet /digital platform? Staff turnover?<br>ii. **If no**, "Please explain why you have not been reporting your service statistics monthly."<br>1. *Probe:* Lack of time to document FP service into the register and monthly summary form? Not comfortable with the DHIS reporting platform? Lack of clients to report on? Challenge with internet /digital platform? Staff turnover?	b. *Probe:* Where did you attend the training?<br>c. *Probe:* How long did the training last? How many hours a day? How many days a week? How many weeks in all?<br>d. *Probe:* What was the cost of the training to you (financial, non-financial)?
	5. How satisfied were you with the structure of the training? Would you have preferred a different structure? Why or why not?<br>a. *Probe*: What fits your lifestyle the best: training in a block (e.g. consecutive days for several weeks) or in a staggered fashion

	(a few days a week for several days or one week at a stretch followed by a break each time, etc.)?<br>b. *Probe:* How satisfied were you with the training overall? Did the training meet your expectations? Please explain. Would you be willing to recommend the training to a colleague? Why or why not?
	6. How valuable was the training to your practice? Please explain to me which parts of the training were most valuable to you and your practice.<br>*a.* *Probe*: How have the knowledge and skills you acquired during the training helped you to improve the services you offer to your clients? *Probe for specific PHC and FP services the training has influenced.*<br>b. *Follow up:* Have you been reporting your service statistics monthly as requested at the training?<br>i. **If yes**, what factors have made it easy for you to report your service statistics?<br>ii. *Probe*: Have you experienced any barriers to regular reporting?<br>1. *Probe:* Lack of time? Not comfortable with the DHIS reporting platform? Other barriers?<br>iii. **If no**, what factors would make it easier for you to report your service statistics?<br>iv. *Probe:* Please explain why you have not been reporting

	your service statistics monthly.<br>1. *Probe:* Lack of time? Not comfortable with the DHIS reporting platform? Other barriers?
5. I would like to ask you a few questions about your perceptions regarding why some CPs did not attend the training on FP organized by IntegratE. Why do you think some of your colleagues did not attend the training?<br>a. *Probe for specific reasons/barriers*: Did they have other engagements over the same period? Were they unwilling to dedicate time to FP training? Were they too busy to leave their pharmacy? Do you think the training was not flexible enough to accommodate their time? Is it because FP is seen as not profitable by pharmacists? **Please explain.**	7. I would like to ask you a few questions about your perceptions regarding why some CPs did not attend the training on FP organized by IntegratE. Why do you think some of your colleagues did not attend the training?<br>a. *Probe for specific reasons/barriers*:<br>i. Scheduling conflicts?<br>ii. Training not seen as profitable?<br>iii. Any other barriers?<br>b. What do you think would encourage these CPs to attend future FP training workshops? Other thoughts?
**CURRENT SUPERVISORY STRUCTURE**<br><br>6. Can you share your experience of supervision engagement with the SMOH, project team and PCN?<br>a. Have you had any experience with the Integrated Supportive Supervision led by the Ministry of Health, and if yes, what has that experience been? *Please note that I am referring to the joint team visits by the SMOH, PCN, Association of Community*	

Pharmacists' leadership and the project team.<br>b. *Probe:* For supervisory services provided – Onsite-training, monitoring visits, data management.<br>c. How effective are these services? And their supervisory systems?	
7. In what ways do the supportive supervision visits support you to improve the quality of services you provide?<br>a. *Probe*: Type of support provided, gaps in provision of support.	
8. In what ways do these organizations (SMOH, PCN, ACPN) constitute barriers to the quality of services you provide?<br>a. *Probe:* Sanctions, shop closure, harassment, limitations to the services you provide?<br>b. *Follow up:* Please tell me about any harassment, sanctions or shop closures affecting you or other CPs you know in your community.	
9. What considerations guide you in stocking commodities? How do you get and manage your drug stock?<br>a. *Probe* for inventory management, drug expiry.	
10. What are your views about the relationship between CPs and PPMVs in your community? To what extent will CPs be open to a closer supervisory relationship with PPMVs?	**PROPOSED SUPERVISORY STRUCTURE**<br>8. What are your views about the relationship between CPs and PPMVs in your community? To what extent will CPs be open to a closer supervisory relationship with PPMVs?

a. *Probe:* To what extent will you, as a CP be open to a closer supervisory relationship with PPMVs?<br>b. *Follow up:* In the proposed hub-and-spoke model, a community pharmacist will supervise or support a group of PPMVs on pharmaceutical care and drug management. Are you open to this model of supervision? Please explain why or why not.<br>c. *Probe:* Please share any personal concerns you may have about such a relationship. *Please remember that all your responses will be kept confidential.*	a. *Probe:* To what extent will you, as a CP be open to a closer supervisory relationship with PPMVs?<br>b. *Follow up:* In the proposed hub-and-spoke model, a community pharmacist will supervise or support a group of PPMVs on pharmaceutical care and drug management. Are you open to this model of supervision? Please explain why or why not.<br>c. *Probe:* Please share any personal concerns you may have about such a relationship. *Please remember that all your responses will be kept confidential.*
**EXPECTATIONS FROM THE ACCREDITATION SYSTEM**<br><br>INT: Thank you for sharing that information with me. I would like to ask you a few questions related to your perceptions of the PCN accreditation system.<br><br>11. What do you know about the proposed accreditation system after the end of IntegratE? (Where PPMVs will be trained by PCN through the School of Health Technology and classified into different tiers based on whether or not they have health qualifications)<br>*a. Probe: Perceived impact on business* (the number of clients, clients' and the community's confidence in services offered, harassment from regulatory and police agencies).<br>b. *Follow up:* What are your thoughts on incorporating the School of Health Technology	**EXPECTATIONS FROM THE ACCREDITATION SYSTEM**<br><br>INT: Thank you for sharing that information with me. I would now like to ask you a few questions related to your perceptions of the PCN accreditation system.<br><br>9. What do you know about the PCN accreditation system where PPMVs are being trained by PCN through the Colleges/Schools of Health Technology and classified into different tiers, based on whether or not they have health qualifications?<br>a. *Follow up:* What are your thoughts on incorporating the School of Health Technology into the tier accreditation system?<br>*b. Probe: Perceived impact on business* (the number of clients, clients' and the community's confidence in services offered,

into the tier accreditation system?	harassment from regulatory and police agencies).
12. What suggestions do you have about how the PCN accreditation system can better improve the quality of family planning services in Nigeria?	10. What suggestions do you have about how the PCN accreditation system can better improve the quality of family planning services in Nigeria?
**ECONOMIC IMPACT OF COVID-19 PANDEMIC**<br>13. As you know, the COVID-19 pandemic has affected different people in different ways. Please explain how the COVID-19 related restrictions have impacted your business.<br>a. *Probe*: How have the COVID-19 related restrictions impacted your personal and family finances?<br>b. *Probe:* What lingering effects has the COVID-19 pandemic had on your client flow?<br>c. *Probe:* What changes, if any, have you noticed in service requests by clients since the pandemic began? Any changes specific to FP service requests? Please explain.	**ECONOMIC IMPACT OF COVID-19 PANDEMIC**<br>11. As you know, the COVID-19 pandemic has affected different people in different ways. Please explain how the COVID-19 related restrictions have impacted you.<br>a. *Probe*: How have the COVID-19 related restrictions affected your business?<br>b. *Probe:* What lingering effects has the COVID-19 pandemic had on your client flow?<br>c. *Probe:* What changes, if any, have you noticed in service requests by clients since the pandemic began? Any changes specific to FP service requests? Please explain.<br>d. *Probe*: How have the COVID-19 related restrictions impacted your personal and family finances?
**Closing**<br><br>Thank you very much for your time and the thoughtful information you have shared with me today!<br><br>Do you have any final comments before we end our discussion?	**Closing**<br><br>Thank you very much for your time and the thoughtful information you have shared with me today!<br><br>Do you have any final comments before we end our discussion?

Supplementary File 2. Interview Guides for Community Pharmacists and Patent and Proprietary Medicine Vendors (below) in Kaduna and Lagos States at Baseline and Follow-Up

**Interview Guide:**<br>**Patent and Proprietary Medicine Vendors**	**Follow Up Interview Guide:**<br>**Patent and Proprietary Medicine Vendors**
**Respondent ID: [__|__|__|__|__|__]**<br><br>**Date:**<br><br>**INTRODUCTION**<br>1. To start, could you tell me a little bit about yourself and your job as a PPMV? What influenced your decision to become a PPMV?	**Respondent ID: [__|__|__|__|__|__]**<br><br>**Date:**<br><br>**INTRODUCTION**<br>**1.** To start, could you tell me a little bit about your job as a PPMV in Lagos/Kaduna state?<br>a. *Follow-up*: When did you enroll with the IntegratE project, and why?<br>b. *Follow-up*: How long have you been providing family planning services?
**CURRENT PRACTICE WITH FAMILY PLANNING**<br>INT: Great, thank you for sharing that with me. I would now like to ask you about your experiences with providing family planning services in your shop.<br><br>2. Can you describe your experience with providing family planning services (products and counselling) in your community?<br>a. *Probe:* In your opinion, what do clients think about the family planning services you provide (women versus men)?<br>b. *Follow-up:* What challenges, if any, have you experienced in providing family planning services in your community? Please explain.	**CURRENT PRACTICE WITH FAMILY PLANNING**<br>INT: Great, thank you for sharing that with me. I would now like to ask you about your experiences with providing family planning services in your shop.<br><br>2. Can you describe your experience with providing family planning services (products and counselling) in your community?<br>a. *Probe:* In your opinion, what do clients think about the family planning services you provide (women versus men)?<br>b. *Follow-up:* What challenges, if any, have you experienced in providing family planning services in your community? Please explain.

i. *Probe:* Specific challenges – cultural, religious, poor client patronage, regulatory, etc.<br>ii. *Preferences:* What have you observed regarding client preferences for specific family planning services? Please elaborate.<br>iii. Are there other methods you wish you could sell or provide? (if yes, please explain)	i. *Probe:* Specific challenges – cultural, religious, poor client patronage, regulatory, etc.<br>ii. *Preferences:* What have you observed regarding client preferences for specific family planning services? Please elaborate.<br>iii. Are there other methods you wish you could sell or provide? (If yes, please explain.)<br>c. *Follow-up*: Beyond family planning services, can you describe your experience with providing primary health care services in your community?<br>i. Are there any other services you provide in your shop? Please explain.
3. In what situations would you refer a client to a health facility or another PPMV?<br>a. *Follow-up:* What factors facilitate referrals for family planning methods?<br>b. *Follow-up:* What do you find difficult about referring clients to a health facility or another PPMV for family planning services?<br>c. *Follow up:* Do you receive feedback from the facility or other PPMVs you refer clients to, or from the clients themselves? Please explain.	3. In what situations would you refer a client to a health facility, or a CP, or another PPMV?<br>a. *Follow-up:* What factors facilitate referrals for family planning methods?<br>b. *Follow-up:* What do you find difficult about referring clients to a health facility or another PPMV for family planning services?<br>c. *Follow up:* Do you receive feedback from the facility or CPs, or other PPMVs you refer clients to, or from the clients themselves? Please explain.
	**RECENT TRAINING AND REPORTING OF SERVICE STATISTICS**<br>4. Please tell me about your experience with the Mandatory Entry Point Training Program.

	a. *Probe:* When did you attend the training?<br>b. *Probe:* Where did you attend the training?<br>c. *Probe:* How long did the training last? How many hours a day? How many days a week? How many weeks in all?<br>d. *Probe:* What was the cost of the training to you (financial, non-financial)?<br><br>5. How satisfied were you with the structure of the training? Would you have preferred a different structure? Why or why not?<br>a. *Probe*: What fits your lifestyle the best: training in a block (e.g. consecutive days for several weeks) or in a staggered fashion (a few days a week for several days or one week at a stretch followed by a break each time, etc.)?<br>b. *Probe:* How satisfied were you with the training overall? Did the training meet your expectations? Please explain. Would you be willing to recommend the training to a colleague? Why or why not?
**FAMILY PLANNING TRAINING AND REPORTING**<br>4. Have you been invited to any training in FP organized by IntegratE? Organized by another organization (*Please ask for name of organization*.)<br>a. *Follow up:* Did you attend the training? How valuable was the training to your practice? Please explain.<br>b. *Follow up:* Have you been reporting your service statistics monthly as requested at the training?	6.How valuable was the training to your practice? Please explain to me which parts of the training were most valuable to you and your practice.<br>a. *Probe*: How have the knowledge and skills you acquired during the training helped you to improve the services you offer to your clients? Probe for specific PHC and FP services the training has influenced.<br>b. *Follow up*: Have you been reporting your service statistics

i. **If yes**, "Have you experienced any barriers to regular reporting?"<br>1. *Probe:* Lack of time to document FP service into the register and monthly summary form? Not comfortable with the DHIS reporting platform? Lack of clients to report on? Challenge with internet /digital platform? Staff turnover?<br>ii. **If no**, "Please explain why you have not been reporting your service statistics monthly."<br>1. *Probe:* Lack of time to document FP service into the register and monthly summary form? Not comfortable with the DHIS reporting platform? Lack of clients to report on? Challenge with internet /digital platform? Staff turnover?	monthly as requested at the training?<br>i. **If yes**, what factors have made it easy for you to report your service statistics?<br>ii. *Follow up*: Have you experienced any barriers to regular reporting?<br>1. *Probe:* Lack of time? Not comfortable with the DHIS reporting platform? Other barriers?<br>iii. **If no**, what factors would make it easier for you to report your service statistics?<br>iv. *Follow up:* Please explain why you have not been reporting your service statistics monthly.<br>2. *Probe:* Lack of time? Not comfortable with the DHIS reporting platform? Other barriers?
5. I would like to ask you a few questions about your perceptions regarding why some PPMVs did not attend the training on FP organized by IntegratE. Why do you think some of your colleagues did not attend the training?<br>a. *Probe for specific reasons/barriers*: Did they have other engagements over the same period? Were they unwilling to dedicate time to FP training? Were they too busy to leave their patent medicine shop? Do you think the training was not flexible enough to accommodate their time? Is it because FP is seen as not	7. I would now like to ask you a few questions about your perceptions regarding why some PPMVs may be reluctant to attend the MEPTP. What should be done to encourage all of your colleagues to attend the training?<br>a. *Probe for specific reasons/barriers*:<br>i. *How may their schedules have affected their decision not to attend the training? Please explain any time constraints you think they may have had*.<br>ii. Please explain how their perception of potential profits from offering family planning services may have

profitable by PPMVs? **Please explain.**	influenced their decision not to attend the training on FP.<br>iii. Please share any logistical or other barriers you think they may have faced, preventing them from attending the FP training.<br>iv. Any other barriers?
	8. What are your thoughts on the current role of the Schools of Health Technology in the training of PPMVs, based on the tier they have been assigned to?<br>a. What suggestions do you have regarding how the involvement of the Schools of Health Technology can better improve the quality of family planning services in Nigeria?
**CURRENT SUPERVISORY STRUCTURE**<br><br>6. Can you share your experience of supervision engagement with the SMOH, project team and PCN?<br>a. Have you had any experience with the Integrated Supportive Supervision led by the Ministry of Health, and if yes, what has that experience been? *Please note that I am referring to the joint team visits by the SMOH, PCN, Association of Community Pharmacists' leadership and the project team.*<br>b. *Probe:* For supervisory services provided – Onsite-training, monitoring visits, data management.<br>c. How effective are these services? And their supervisory systems?	

7. In what ways do the supportive supervision visits support you to improve the quality of services you provide?<br>a. Probe: Type of support provided, gaps in provision of support.	
8. In what ways do these organizations (SMOH, PCN, ACPN) constitute barriers to the quality of services you provide?<br>a. *Probe:* Sanctions, shop closure, harassment, limitations to the services you provide?<br>b. *Follow up:* Please tell me about any harassment, sanctions or shop closures affecting you or other PPMVs you know in your community.	
9. What considerations guide you in stocking commodities? How do you get and manage your drug stock?<br>a. *Probe* for inventory management, drug expiry.<br>b. *Probe:* What about family planning commodities in particular?<br>c. *Follow up:* What challenges, if any, do you face in stocking drugs? In stocking FP commodities in particular?	
10. What are your views about the relationship between PPMVs and pharmacists in your community? To what extent will PPMVs be open to a closer business relationship with CPs?<br>a. *Probe:* To what extent will you, as a PPMV be open to a closer	**PROPOSED SUPERVISORY STRUCTURE**<br>9. What are your views about the relationship between PPMVs and pharmacists in your community? To what extent will PPMVs be open to a closer business relationship with CPs?

supervisory relationship with CPs?<br>b. *Follow up:* In the proposed hub-and-spoke model, a community pharmacist will supervise or support a group of PPMVs on pharmaceutical care and drug management. Are you open to this model of supervision? Please explain why or why not.<br>*c.* Please share any personal concerns you may have about such a relationship. *Please remember that all your responses will be kept confidential.*	a. *Probe:* To what extent will you, as a PPMV be open to a closer supervisory relationship with CPs?<br>b. *Follow up:* In the proposed hub-and-spoke model, a community pharmacist will supervise or support a group of PPMVs on pharmaceutical care and drug management. Are you open to this model of supervision? Please explain why or why not.<br>*c.* Please share any personal concerns you may have about such a relationship. *Please remember that all your responses will be kept confidential.*
**EXPECTATIONS FROM THE ACCREDITATION SYSTEM**<br>INT: Thank you for sharing that information with me. I would like to ask you a few questions related to your expectations about the PCN accreditation system.<br>*11.* What do you know about the proposed accreditation system after the end of IntegratE? (Where PPMVs will be trained by PCN through the School of Health Technology and classified into different tiers based on whether or not they have health qualifications)<br>*a. Probe: Perceived impact on business* (the number of clients, clients' and the community's confidence in services offered, harassment from regulatory and police agencies).<br>b. *Follow up:* What are your thoughts on incorporating the School of Health Technology into the tier accreditation system?	

c. Probe: Have you registered for the PCN accreditation training – Mandatory Entry Point Training Program (MEPTP)? Why or why not? Do you know of anyone who has registered? Please explain.	
12. What suggestions do you have about how the PCN accreditation system can better improve the quality of family planning services in Nigeria?	
**ECONOMIC IMPACT OF COVID-19 PANDEMIC**<br>13. As you know, the COVID-19 pandemic has affected different people in different ways. Please explain how the COVID-19 related restrictions have impacted your business.<br>a. *Probe*: How have the COVID-19 related restrictions impacted your personal and family finances?<br>b. *Probe:* What lingering effects has the COVID-19 pandemic had on your client flow?<br>c. *Probe:* What changes, if any, have you noticed in service requests by clients since the pandemic began? Any changes specific to FP service requests? Please explain.	**ECONOMIC IMPACT OF COVID-19 PANDEMIC**<br>10. As you know, the COVID-19 pandemic has affected different people in different ways. Please explain how the COVID-19 related restrictions have impacted you.<br>a. *Probe*: How have the COVID-19 related restrictions affected your business?<br>b. *Probe:* What lingering effects has the COVID-19 pandemic had on your client flow?<br>c. *Probe:* What changes, if any, have you noticed in service requests by clients since the pandemic began? Any changes specific to FP service requests? Please explain.<br>d. *Probe*: How have the COVID-19 related restrictions impacted your personal and family finances?
**Closing**<br><br>Thank you very much for your time and the thoughtful information you have shared with me today!<br><br>Do you have any final comments before we end our discussion?	**Closing**<br><br>Thank you very much for your time and the thoughtful information you have shared with me today!<br><br>Do you have any final comments before we end our discussion?
